# High-sensitivity troponin in the evaluation of patients with suspected acute coronary syndrome: a stepped-wedge, cluster-randomised controlled trial

**DOI:** 10.1016/S0140-6736(18)31923-8

**Published:** 2018-09-15

**Authors:** Anoop S V Shah, Atul Anand, Fiona E Strachan, Amy V Ferry, Kuan Ken Lee, Andrew R Chapman, Dennis Sandeman, Catherine L Stables, Philip D Adamson, Jack P M Andrews, Mohamed S Anwar, John Hung, Alistair J Moss, Rachel O'Brien, Colin Berry, Iain Findlay, Simon Walker, Anne Cruickshank, Alan Reid, Alasdair Gray, Paul O Collinson, Fred S Apple, David A McAllister, Donogh Maguire, Keith A A Fox, David E Newby, Christopher Tuck, Ronald Harkess, Richard A Parker, Catriona Keerie, Christopher J Weir, Nicholas L Mills, High-STEACS Investigators, Lucy Marshall, Stacey D Stewart, Takeshi Fujisawa, Catalina A Vallejos, Athanasios Tsanas, Mischa Hautvast, Jean McPherson, Lynn McKinlay, Jonathan Malo, Colin M Fischbacher, Bernard L Croal, Stephen J Leslie, Allan Walker, Tony Wackett, Roma Armstrong, Laura Stirling, Claire MacDonald, Imran Sadat, Frank Finlay, Heather Charles, Pamela Linksted, Stephen Young, Bill Alexander, Chris Duncan

**Affiliations:** a British Heart Foundation Centre for Cardiovascular Science, University of Edinburgh, Edinburgh, UK; b Usher Institute of Population Health Sciences and Informatics, University of Edinburgh, Edinburgh, UK; c Edinburgh Clinical Trials Unit, University of Edinburgh, Edinburgh, UK; d Department of Cardiology, Victoria Hospital, Kirkcaldy, UK; e Emergency Medicine Research Group of Edinburgh, Royal Infirmary of Edinburgh, Edinburgh, UK; f Department of Clinical Biochemistry, Royal Infirmary of Edinburgh, Edinburgh, UK; g Institute of Cardiovascular and Medical Sciences, University of Glasgow, Glasgow, UK; h Institute of Health and Wellbeing, University of Glasgow, Glasgow, UK; i Department of Cardiology, Royal Alexandra Hospital, Paisley, UK; j Department of Biochemistry, Queen Elizabeth University Hospital, Glasgow, UK; k Department of Clinical Blood Sciences and Department of Cardiology, St George's University Hospitals NHS Trust and St George's University of London, London, UK; l Department of Laboratory Medicine and Pathology, Hennepin County Medical Center and University of Minnesota, Minneapolis, MN, USA; m Emergency Medicine Department, Glasgow Royal Infirmary, Glasgow, UK

## Abstract

**Background:**

High-sensitivity cardiac troponin assays permit use of lower thresholds for the diagnosis of myocardial infarction, but whether this improves clinical outcomes is unknown. We aimed to determine whether the introduction of a high-sensitivity cardiac troponin I (hs-cTnI) assay with a sex-specific 99th centile diagnostic threshold would reduce subsequent myocardial infarction or cardiovascular death in patients with suspected acute coronary syndrome.

**Methods:**

In this stepped-wedge, cluster-randomised controlled trial across ten secondary or tertiary care hospitals in Scotland, we evaluated the implementation of an hs-cTnI assay in consecutive patients who had been admitted to the hospitals' emergency departments with suspected acute coronary syndrome. Patients were eligible for inclusion if they presented with suspected acute coronary syndrome and had paired cardiac troponin measurements from the standard care and trial assays. During a validation phase of 6–12 months, results from the hs-cTnI assay were concealed from the attending clinician, and a contemporary cardiac troponin I (cTnI) assay was used to guide care. Hospitals were randomly allocated to early (n=5 hospitals) or late (n=5 hospitals) implementation, in which the high-sensitivity assay and sex-specific 99th centile diagnostic threshold was introduced immediately after the 6-month validation phase or was deferred for a further 6 months. Patients reclassified by the high-sensitivity assay were defined as those with an increased hs-cTnI concentration in whom cTnI concentrations were below the diagnostic threshold on the contemporary assay. The primary outcome was subsequent myocardial infarction or death from cardiovascular causes at 1 year after initial presentation. Outcomes were compared in patients reclassified by the high-sensitivity assay before and after its implementation by use of an adjusted generalised linear mixed model. This trial is registered with ClinicalTrials.gov, number NCT01852123.

**Findings:**

Between June 10, 2013, and March 3, 2016, we enrolled 48 282 consecutive patients (61 [SD 17] years, 47% women) of whom 10 360 (21%) patients had cTnI concentrations greater than those of the 99th centile of the normal range of values, who were identified by the contemporary assay or the high-sensitivity assay. The high-sensitivity assay reclassified 1771 (17%) of 10 360 patients with myocardial injury or infarction who were not identified by the contemporary assay. In those reclassified, subsequent myocardial infarction or cardiovascular death within 1 year occurred in 105 (15%) of 720 patients in the validation phase and 131 (12%) of 1051 patients in the implementation phase (adjusted odds ratio for implementation *vs* validation phase 1·10, 95% CI 0·75 to 1·61; p=0·620).

**Interpretation:**

Use of a high-sensitivity assay prompted reclassification of 1771 (17%) of 10 360 patients with myocardial injury or infarction, but was not associated with a lower subsequent incidence of myocardial infarction or cardiovascular death at 1 year. Our findings question whether the diagnostic threshold for myocardial infarction should be based on the 99th centile derived from a normal reference population.

**Funding:**

The British Heart Foundation.

## Introduction

Myocardial infarction is defined by the clinical history, electrocardiogram, and an increase or decrease in cardiac troponin concentration (as evidence of myocardial necrosis).^1^ Improvements in assay sensitivity now permit the quantification of very low concentrations of troponin with high precision, which allows the use of lower diagnostic thresholds.^2^ The Universal Definition of Myocardial Infarction^1^ recommends that an increase in troponin above the 99th centile of a normal reference population should be used as the threshold for diagnosis of myocardial infarction. Furthermore, it recognises that troponin concentrations differ in men and women,^3^, ^4^ and suggests sex-specific diagnostic thresholds be applied when using high-sensitivity assays.

**Research in context**

**Evidence before this study**

We searched PubMed for reports published in English between Jan 1, 2010, and July 18, 2018, with the search terms “cardiac troponin”, “myocardial infarction”, “acute coronary syndrome”, and “randomised controlled trials”. Although no randomised controlled trials have evaluated the effects of a high-sensitivity cardiac troponin assay on cardiovascular outcomes, a previous study showed that lowering the diagnostic threshold with a contemporary troponin assay was associated with a reduction in myocardial infarction or death in those reclassified as having had a myocardial infarction. Further, registry studies have shown a reduction in recurrent myocardial infarction but no difference in all-cause mortality following the introduction of a high-sensitivity cardiac troponin assay.

**Added value of this study**

To our knowledge, this is the first randomised controlled trial to evaluate the effects of implementing a high-sensitivity cardiac troponin I assay in patients with suspected acute coronary syndrome. We show that use of the high-sensitivity assay reclassified one in six patients with myocardial injury, but only a third of these patients had a diagnosis of type 1 myocardial infarction, and the incidence of subsequent myocardial infarction or cardiovascular death at 1 year was unchanged.

**Implications of all the available evidence**

Implementation of a high-sensitivity cardiac troponin assay and use of the 99th centile as the diagnostic threshold identifies more patients with myocardial injury than type 1 myocardial infarction and does not lead to a reduction in subsequent cardiac events. This finding raises the question of whether clinical decisions should be based on a 99th centile threshold derived from a reference population or on an approach that acknowledges the continuum of disease and clinical presentation and optimises diagnostic accuracy. Finally, a high-sensitivity assay can identify low-risk populations, leading to reductions in the overall duration of hospital stay.

The use of high-sensitivity cardiac troponin assays and lowering the diagnostic threshold to the 99th centile remains a contentious issue in clinical practice;^5^ therefore, despite guideline recommendations,^1^ few institutions worldwide have adopted high-sensitivity assays.^6^, ^7^ If increased sensitivity does not affect the specificity of testing for the diagnosis of myocardial infarction, then introducing high-sensitivity assays will improve patient outcomes through better targeting of therapies for coronary heart disease. However, if the increase in sensitivity leads to poor specificity, then patients could be misdiagnosed, given inappropriate medications, and potentially have adverse outcomes. We aimed to determine whether the introduction of a high-sensitivity cardiac troponin I (hs-cTnI) assay with a sex-specific 99th centile diagnostic threshold would reduce subsequent myocardial infarction or cardiovascular death within 1 year in patients with suspected acute coronary syndrome who would previously have been classified as not having had a myocardial injury and were reclassified following use of the high-sensitivity assay.

## Methods

### Study design and participants

The High-Sensitivity Troponin in the Evaluation of patients with suspected Acute Coronary Syndrome (High-STEACS) trial is a stepped-wedge, cluster-randomised controlled trial that aimed to prospectively evaluate the implementation of an hs-cTnI assay in consecutive patients with suspected acute coronary syndrome in ten secondary and tertiary care hospitals in Scotland. Sites were eligible if they had the capacity to measure the trial assay and if they returned data to the national hospital admissions database.^8^ All patients attending the Emergency Department were screened for suspected acute coronary syndrome by the attending clinician; at the same time, troponin was requested with an electronic form integrated into the clinical care pathway. Patients were eligible for inclusion if they presented with suspected acute coronary syndrome and had paired cardiac troponin measurements from the standard care and trial assays. Patients were excluded if they had been admitted previously during the trial period or were not resident in Scotland.

**Figure 1:**
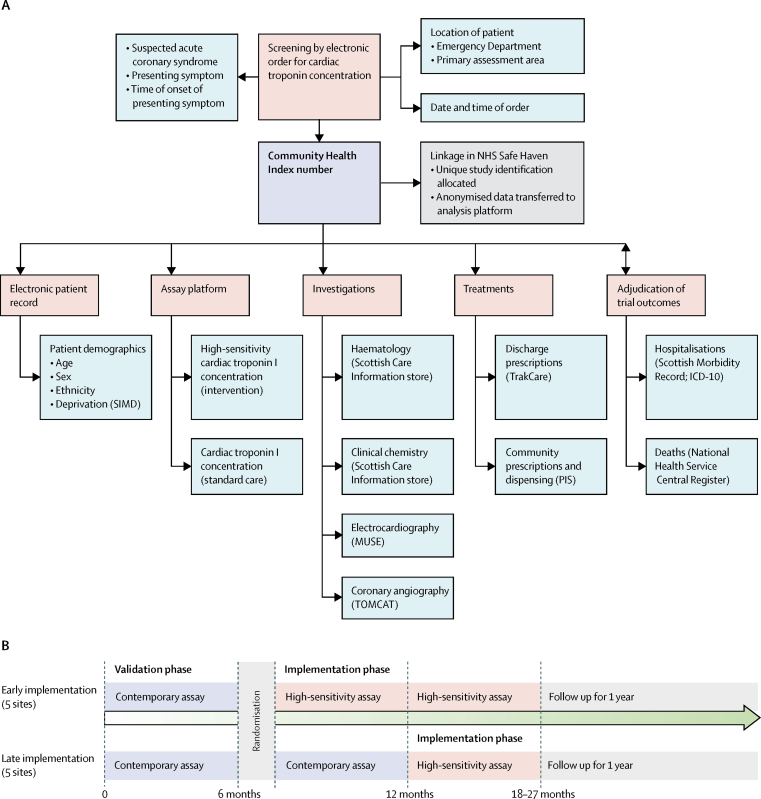
Schematic of the High-STEACS trial design and linkage of electronic patient record data sources (A) Diagram illustrating how screening, enrolment, adjudication, and follow-up were done by use of linked routine health-care data in Scotland. The Community Health Index is a population health-care register that includes all individuals resident in Scotland. The Community Health Index number, date and time of presentation, and study inclusion and exclusion criteria were extracted from the TrakCare software application and linked to the ARCHITECT assay platform to identify eligible patients. This number was also used to link all data sources, which are held securely within the NHS safe haven of each Health Board. Eligible patients were assigned a unique study ID and all identifiable data were removed. Anonymised data were transferred to a national analytical platform in the Farr Institute of Health Informatics Research (Edinburgh Bioquarter) for analysis and reporting. (B) Study design, in which sites were separated into early and late implementation designs. ICD-10=International Classification of Diseases, tenth edition. PIS=Prescribing Information System. SIMD=Scottish Index of Multiple Deprivation.

The study was approved by the Scotland A Research Ethics Committee, the Public Benefit and Privacy Panel for Health and Social Care, and by each National Health Service (NHS) Health Board. The conduct of the trial was periodically reviewed by an independent data monitoring committee. All data were collected prospectively from the electronic patient record, de-identified and linked within secure NHS Safe Havens (figure 1).

### Randomisation and masking

In this trial, the hospital site was the unit of randomisation. Cluster randomisation was necessary to avoid the risk of clinical error due to reporting of different troponin assays and thresholds simultaneously. All sites reported cardiac troponin concentration by use of a contemporary troponin assay and an existing diagnostic threshold in a validation phase of at least 6 months. Sites were paired based on expected number of patients presenting with suspected acute coronary syndrome before they were randomly allocated to early or late implementation of the high-sensitivity assay (with sex-specific thresholds) for the diagnosis of myocardial infarction (figure 1; appendix). Allocation was masked from sites before their inclusion in the trial and allocation was masked from individual participants throughout.

### Procedures

Cardiac troponin testing was done when patients presented at the hospital and was repeated 6 or 12 hours after the onset of symptoms, at the discretion of the attending physician and in accordance with national and international guidelines.^9^, ^10^ Throughout the trial period, contemporary and high-sensitivity troponin assays were run simultaneously in plasma that had been taken but was surplus to clinical requirements. Attending clinicians were masked to the results of the high-sensitivity assay during the validation phase and the contemporary assay during the implementation phase.

In the validation phase, a contemporary cardiac troponin I (cTnI) assay (Abbott Laboratories; Abbott Park, IL, USA) was used to guide clinical decisions. The inter-assay coefficient of variation was determined at each site and was less than 10% at 40 ng/L (seven sites) and 50 ng/L (three sites). Only cTnI concentrations above these diagnostic thresholds were reported.^11^ During the implementation phase, an hs-cTnI assay (ARCHITECT_STAT_ high-sensitive troponin I assay; Abbott Laboratories) was used to guide clinical decisions. This assay has an interassay coefficient of variation of less than 10% at 4·7 ng/L,^12^ and a 99th centile upper reference limit of 34 ng/L in men and 16 ng/L in women.^3^ To support implementation, we provided written educational material and presentations at each site and training for clinical and laboratory staff, and we updated the electronic patient record to highlight the change in assay and diagnostic thresholds.

We collected clinical information from a standardised electronic patient record (TrakCare; InterSystems Corporation, Cambridge, MA, USA). All patients with hs-cTnI concentrations above the 99th centile were assessed in accordance with the Universal Definition of Myocardial Infarction,^1^ as previously described.^13^, ^14^ Two physicians who were masked to the study phase independently reviewed all clinical information, and discordant diagnoses were resolved by a third reviewer. Type 1 myocardial infarction was defined as myocardial necrosis (any hs-cTnI concentration above the 99th centile with an increase or decrease in hs-cTnI concentration, where serial testing was done) in the context of a presentation with suspected acute coronary syndrome, with symptoms or signs of myocardial ischaemia on an electrocardiogram. Symptoms or signs of myocardial ischaemia due to increased oxygen demand or decreased oxygen supply (for example, tachyarrhythmia, hypotension, or anaemia) secondary to an alternative pathology and myocardial necrosis were defined as type 2 myocardial infarction. Type 4a myocardial infarction was defined in patients with symptoms or signs of myocardial ischaemia following percutaneous coronary intervention, where hs-cTnI concentrations were 5 times greater than the 99th centile, or when concentrations had increased further if they were increased before the procedure. Type 4b myocardial infarction was defined as myocardial ischaemia and myocardial necrosis that was associated with stent thrombosis, documented at angiography. Myocardial injury was defined as hs-cTnI concentrations greater than the 99th centile of normal reference values in the absence of any clinical features of myocardial ischaemia.

The study population was stratified by peak troponin concentration. Patients with hs-cTnI concentrations within the reference range (1–16 ng/L in women, 1–34 ng/L in men) were classified as having no myocardial injury. Patients with myocardial injury identified by the contemporary assay were defined as those with any cTnI concentration greater the diagnostic threshold of this assay. Patients reclassified by the hs-cTnI assay were defined as those with an increased hs-cTnI concentration (>16 ng/L for women, >34 ng/L for men), in whom cTnI concentrations were below the diagnostic threshold on the contemporary assay.

### Outcomes

We used regional and national registries to ensure complete follow-up for the trial population^12^, ^13^, ^14^, ^15^ (figure 1; appendix). The prespecified primary outcome was subsequent myocardial infarction (type 1 or type 4b) or cardiovascular death within 1 year following the initial presentation to hospital. Primary outcome events were adjudicated by investigators who were masked to troponin concentrations during the index (ie, initial) presentation and study phase. The secondary efficacy outcomes were duration of hospital stay, myocardial infarction (type 1 or type 4b), unplanned coronary revascularisation, all-cause death, death from cardiovascular causes (cardiac and non-cardiac), hospital admission for heart failure, and ischaemic stroke. Secondary safety outcomes were major haemorrhage, unplanned hospital admission, excluding acute coronary syndrome, and non-cardiovascular death.

### Statistical analysis

We estimated that 6·4% of patients would be reclassified by the high-sensitivity assay^3^ and that the event rate for the primary outcome would be 13% in this group.^11^ Based on the planned inclusion of ten sites, power was 74–85% for an absolute risk reduction of 4·4%, if the proportion reclassified was between 6% and 9%, and the intra-cluster correlation coefficient was between 0·05 and 0·10 (appendix). Outcomes were compared in patients who had been reclassified by the hs-cTnI assay before and after its implementation by use of a generalised linear mixed effects model; the effects of the intervention were presented as odds ratios (ORs) with 95% CIs. The model adjusted for site, season, and time of presentation from the start date of the trial. Hospital site was fitted as a random effect, and age, sex, and social deprivation were included as fixed patient-level covariates. In a sensitivity analysis, an additional random effect was included in the primary analysis model to test for site-by-intervention interaction. Outcome event times were summarised descriptively before and after implementation of the high-sensitivity assay by use of Kaplan-Meier survival curves, and differences between phases were tested with a log-rank test. Statistical analysis was done with SAS version 9.4. This trial is registered with ClinicalTrials.gov, number NCT01852123.

### Role of the funding source

The funders of the study had no role in study design, data collection, data analysis, data interpretation, or writing of the report. The corresponding author had full access to all the data in the study and had final responsibility for the decision to submit for publication.

## Results

Ten of the 23 secondary or tertiary care hospitals in Scotland were eligible and all of these ten hospitals participated (appendix). Between June 10, 2013, and March 3, 2016, 48 282 consecutive patients with suspected acute coronary syndrome (61 [SD 17] years, 47% women) met the trial inclusion criteria (appendix) and were included in the analysis of the primary outcome. The trial concluded on March 3, 2017, after a minimum follow-up period of 1 year. 18 978 (39%) patients were admitted during the validation phase and 29 304 (61%) patients were admitted during the implementation phase. 32 045 (66%) patients were admitted across sites assigned to the early implementation group and 16 237 (34%) patients were admitted across sites assigned to the late implementation group.

Baseline characteristics of the study population are summarised in table 1, stratified by phase and analysis population. The study population was stratified by peak troponin concentration. During the initial presentation to hospital, we identified 10 360 (21%) of these 48 282 patients with hs-cTnI concentrations greater than the 99th centile of normal reference values. Of the 10 360 patients with increased hs-cTnI concentrations, 1771 (17%) patients were reclassified by the high-sensitivity assay and 8589 (83%) patients were identified by the contemporary assay (table 1). Patients reclassified were older (75 [SD 14] years in the reclassified group *vs* 70 [15] years in those identified by the contemporary assay) and twice as likely to be women (83% *vs* 41%) than those identified by the contemporary assay. Compared with patients identified by the contemporary assay, those reclassified were as likely to present with chest pain (67% in those reclassified *vs* 71% in those identified by the cTn I assay) and have a history of ischaemic heart disease (36% *vs* 33%), but were less likely to show myocardial ischaemia on the electrocardiograph (14% *vs* 36%). Clinical characteristics (such as presenting symptoms and comorbidities) were similar in each analysis population across both phases (appendix).

**Table 1:** Characteristics of the trial participants, stratified by troponin concentration

		**All participants**	**No myocardial injury**	**Myocardial injury**	
				Reclassified by high-sensitivity cardiac troponin I assay	Identified by cardiac troponin I assay
Number of participants		48 282	37 922	1771	8589
Age, years		61 (17)	58 (17)	75 (14)	70 (15)
Sex					
	Women	22 562 (47%)	17 571 (46%)	1470 (83%)	3521 (41%)
	Men	25 720 (53%)	20 351 (54%)	301 (17%)	5068 (59%)
Phase					
	Validation	18 978 (39%)	14 862 (39%)	720 (41%)	3396 (40%)
	Implementation	29 304 (61%)	23 060 (61%)	1051 (59%)	5193 (60%)
Presenting complaint*					
	Chest pain	34 540 (81%)	28 091 (84%)	1074 (67%)	5375 (71%)
	Dyspnoea	2175 (5%)	1107 (3%)	202 (13%)	866 (11%)
	Palpitation	1269 (3%)	991 (3%)	72 (4%)	206 (3%)
	Syncope	2495 (6%)	1809 (5%)	125 (8%)	561 (7%)
	Other	2188 (5%)	1458 (4%)	128 (8%)	602 (8%)
Previous medical conditions					
	Myocardial infarction	4214 (9%)	2835 (7%)	219 (12%)	1160 (14%)
	Ischaemic heart disease	11 912 (25%)	8455 (22%)	645 (36%)	2812 (33%)
	Cerebrovascular disease	2949 (6%)	1915 (5%)	210 (12%)	824 (10%)
	Diabetes mellitus	3518 (7%)	2040 (5%)	218 (12%)	1260 (15%)
Previous revascularisation					
	Percutaneous coronary intervention	3682 (8%)	2744 (7%)	155 (9%)	783 (9%)
	Coronary artery bypass grafting	782 (2%)	534 (1%)	40 (2%)	208 (2%)
Medications at presentation					
	Aspirin	13 163 (27%)	9462 (25%)	668 (38%)	3033 (35%)
	Dual anti-platelet therapy†	1605 (3%)	1103 (3%)	88 (5%)	414 (5%)
	Statin	19 366 (40%)	14 106 (37%)	960 (54%)	4300 (50%)
	Angiotensin converting enzyme inhibitor or angiotensin receptor blockers	15 618 (32%)	11 285 (30%)	762 (43%)	3571 (42%)
	Beta-blocker	13 173 (27%)	9566 (25%)	658 (37%)	2949 (34%)
	Oral anticoagulant‡	3253 (7%)	2158 (6%)	238 (13%)	857 (10%)
Electrocardiogram result§					
	Normal	..	..	592 (43%)	2080 (32%)
	Myocardial ischaemia	..	..	194 (14%)	2316 (36%)
	ST segment elevation	..	..	32 (2%)	966 (15%)
	ST segment depression	..	..	125 (9%)	1203 (19%)
	Left bundle branch block	..	..	30 (2%)	157 (2%)
	T wave inversion	..	..	192 (14%)	1085 (17%)
Physiological parameters					
	Heart rate, beats per minute	..	..	86 (27)	86 (26)
	Systolic blood pressure, mm Hg	..	..	143 (28)	138 (29)
	GRACE risk score	..	..	141 (32)	144 (39)
Haematology and clinical chemistry measurements					
	Haemoglobin, g/L	136 (22)	137 (20)	126 (22)	132 (25)
	Estimated glomerular filtration rate, mL/min	54 (13)	56 (10)	47 (15)	48 (16)
	Peak high-sensitivity cardiac troponin I, ng/L	4 (2–16)	3 (1–6)	26 (20–37)	297 (76–2600)
	Serial high-sensitivity cardiac troponin I testing¶	23 011 (48%)	16 028 (42%)	1024 (58%)	5959 (69%)

Data are number of patients (%), mean (SD), or median (IQR). GRACE=Global Registry of Acute Coronary Events.

* A presenting symptom was missing in 5615 (12%) patients.

† Two medications from aspirin, clopidogrel, prasugrel, or ticagrelor.

‡ Includes warfarin or novel oral anticoagulants.

§ Electrocardiogram data were available in 1377 (78%) of reclassified patients and 6470 (75%) of identified patients.

¶ Defined as two or more tests within 24 hours from presentation.

**Figure 2:**
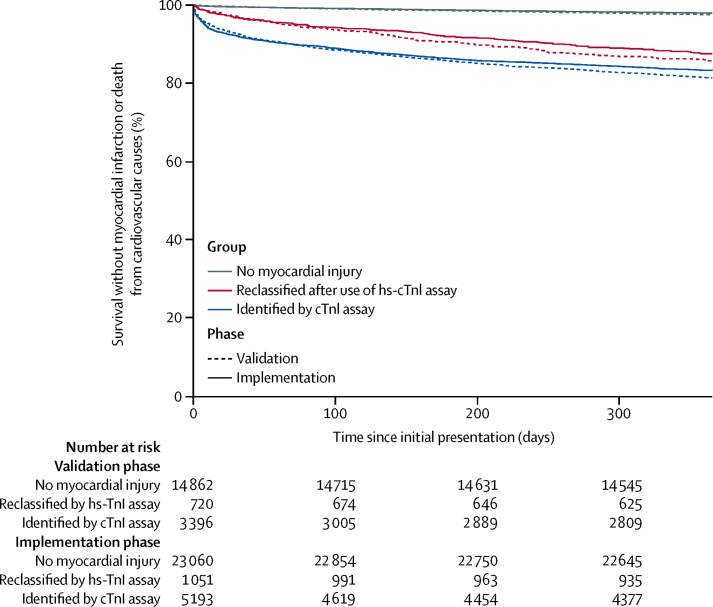
Incidence of myocardial infarction or death from cardiovascular causes at 1 year, stratified by troponin concentration and phase Data are Kaplan-Meier time-to-event curves. Paired log-rank test results are p=0·047 for no myocardial injury, p=0·131 for those reclassified by the hs-cTnI assay, and p=0·019 for those already identified by the contemporary cTnI assay. hs-cTnI=high-sensitivity cardiac troponin I. cTnI=contemporary cardiac troponin I.

**Figure 3:**
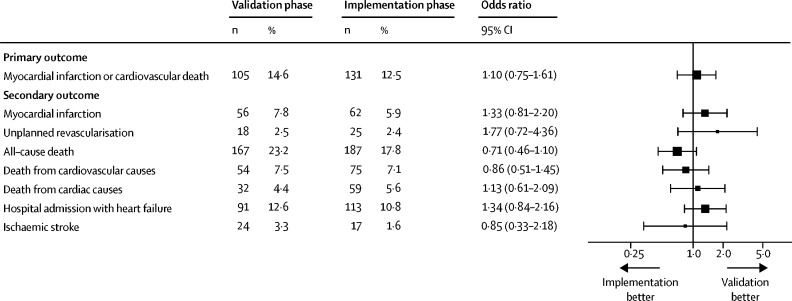
Primary and secondary outcomes in patients reclassified by the high-sensitivity cardiac troponin I assay before and after implementation Data are the number and percentage of patients with each outcome in the validation phase and implementation phase and the odds ratio for implementation versus validation. The intra-cluster correlation coefficient from the generalised linear mixed effects model was 0.

Patients were followed up for 1 year for all primary and secondary outcome measures. Within 1 year, 2586 (5%) of 48 282 patients with suspected acute coronary syndrome had a subsequent myocardial infarction or death from cardiovascular causes (table 2). The primary outcome occurred in 1106 (6%) of 18 978 patients who presented during the validation phase and in 1480 (5%) of 29 304 patients who presented during the implementation phase (adjusted OR for implementation *vs* validation phase 1·05, 95% CI 0·92–1·19; p=0·48). Patients with myocardial injury were more likely than those without to have a subsequent myocardial infarction or death from cardiovascular causes within 1 year (figure 2). In patients who were reclassified by the high-sensitivity assay, the primary outcome occurred in 105 (15%) of 720 patients during the validation phase and 131 (12%) of 1051 patients in the implementation phase (1·10, 0·75–1·61; p=0·620; figure 3). In reclassified patients, there were no differences in any of the secondary efficacy and safety outcome measures between phases (table 2; figure 3).

**Table 2:** Primary and secondary outcomes after 1 year in participants, stratified by troponin concentration and phase

		**All participants (n=48 282)**	**No myocardial injury**		**Myocardial injury**			
			Validation (n=14 862)	Implementation (n=23 060)	Reclassified by high-sensitivity cardiac troponin I assay		Identified by cardiac troponin I assay	
					Validation (n=720)	Implementation (n=1051)	Validation (n=3396)	Implementation (n=5193)
**Primary outcome**								
Myocardial infarction* or death from cardiovascular causes		2586 (5%)	367 (2%)	479 (2%)	105 (15%)	131 (12%)	634 (19%)	870 (17%)
**Secondary outcomes**								
Myocardial infarction*		1046 (2%)	163 (1%)	198 (1%)	56 (8%)	62 (6%)	249 (7%)	318 (6%)
Unplanned revascularisation†		672 (1%)	80 (1%)	182 (1%)	18 (3%)	25 (2%)	147 (4%)	220 (4%)
All-cause death		4367 (9%)	824 (6%)	1170 (5%)	167 (23%)	187 (18%)	882 (26%)	1137 (22%)
	Death from cardiovascular causes	1693 (4%)	217 (1%)	299 (1%)	54 (8%)	75 (7%)	432 (13%)	616 (12%)
	Death from cardiac causes	1273 (3%)	143 (1%)	191 (1%)	32 (4%)	59 (6%)	349 (10%)	499 (10%)
Hospital admission for heart failure		1700 (4%)	334 (2%)	337 (1%)	91 (13%)	113 (11%)	371 (11%)	454 (9%)
Ischaemic stroke		546 (1%)	171 (1%)	173 (1%)	24 (3%)	17 (2%)	78 (2%)	83 (2%)
**Safety endpoints**								
Major haemorrhage‡		195 (<1%)	40 (<1%)	55 (<1%)	5 (1%)	11 (1%)	38 (1%)	46 (1%)
Unplanned hospital admission at 30 days§		8489 (18%)	2450 (17%)	2995 (13%)	208 (29%)	245 (23%)	1190 (35%)	1401 (27%)
Non-cardiovascular death		2673 (6%)	607 (4%)	871 (4%)	113 (16%)	111 (11%)	450 (13%)	521 (10%)

Data are number of patients (%).

* Subsequent type 1 or type 4b myocardial infarction.

† Defined as urgent or emergency percutaneous coronary intervention or coronary artery bypass grafting from discharge to 1 year later.

‡ Bleeding Academic Research Consortium type 3 or type 5.

§ Excludes type 1 or type 4b myocardial infarction.

The index diagnosis was adjudicated in all patients with hs-cTnI concentrations greater than the 99th centile. Where a consensus was reached by the adjudication panel in 9115 (88%) patients, the diagnosis was type 1 myocardial infarction in 5028 (55%) patients, type 2 myocardial infarction in 1260 (14%) patients, and myocardial injury in 2810 (31%) patients. Compared with patients identified by the contemporary assay, those reclassified by the high-sensitivity assay were less likely to have type 1 myocardial infarction (515 [33%] patients who had been reclassified *vs* 4513 [60%] patients who had been identified by the contemporary assay), and more likely to be classified as having myocardial injury (796 [51%] patients *vs* 2014 [27%] patients).

Management during the index presentation was compared. Patients reclassified by the high-sensitivity assay were more likely to undergo coronary angiography in the implementation phase compared with the validation phase (11% in the implementation phase *vs* 4% in the validation phase), but percutaneous coronary intervention (5% *vs* 3%) did not differ (table 3). Similarly, there were more new prescriptions for additional anti-platelet therapy (18% *vs* 9%) and other secondary prevention agents during the implementation phase than the validation phase. The duration of hospital stay was longer in the implementation phase than the validation phase in reclassified patients (median 51 h, IQR 20–134 in the implementation phase; *vs* 21 h, 4–101 in the validation phase), but was shorter in patients without myocardial injury (4 h, 3–20; *vs* 7 h, 3–24) and in the study population overall (8 h, 3–40; *vs* 11 h, 4–38; table 3).

**Table 3:** Management of patients during initial hospital admission, stratified by troponin concentration and phase

	**No myocardial injury (n=37 922)**		**Myocardial injury**			
	Validation	Implementation	Reclassified by high-sensitivity cardiac troponin I assay (n=1771)		Identified by cardiac troponin I assay (n=8589)	
			Validation	Implementation	Validation	Implementation
Number of participants	14 862 (39%)	23 060 (61%)	720 (41%)	1051 (59%)	3396 (40%)	5193 (60%)
Duration of hospital stay, h	7 (3–24)	4 (3–20)	21 (4–101)	51 (20–134)	82 (19–186)	78 (37–164)
Coronary angiography*	204 (1%)	329 (1%)	29 (4%)	111 (11%)	1108 (33%)	2177 (42%)
Percutaneous coronary intervention or coronary artery bypass grafting	112 (1%)	187 (1%)	23 (3%)	51 (5%)	706 (21%)	1535 (30%)
New anti-platelet drug	795 (5%)	976 (4%)	64 (9%)	194 (18%)	1408 (41%)	2428 (47%)
New dual anti-platelet therapy†	248 (2%)	336 (1%)	35 (5%)	124 (12%)	1144 (34%)	2080 (40%)
New statin therapy	419 (3%)	608 (3%)	32 (4%)	79 (8%)	660 (19%)	1263 (24%)
New angiotensin converting enzyme inhibitor or angiotensin receptor blocker	287 (2%)	479 (2%)	34 (5%)	77 (7%)	671 (20%)	1163 (22%)
New beta-blocker	765 (5%)	1092 (5%)	65 (9%)	164 (16%)	828 (24%)	1502 (29%)

Data are number of patients (%) or median (IQR).

* Angiography and revascularisation within 30 days of presentation.

† Two medications from aspirin, clopidogrel, prasugrel, or ticagrelor.

## Discussion

We evaluated whether the use of a high-sensitivity cardiac troponin assay was beneficial or harmful in 48 282 consecutive patients with suspected acute coronary syndrome. Introduction of the high-sensitivity assay prompted reclassification of 1771 (17%) patients with myocardial injury; however, only a third of these patients had a diagnosis of type 1 myocardial infarction and the incidence of subsequent myocardial infarction or death from cardiovascular causes within 1 year was not changed by introduction of this high-sensitivity assay.

There are several strengths of our trial design.^16^ First, we enrolled consecutive patients in whom the attending clinician suspected acute coronary syndrome by embedding our screening tool into the electronic health-care system. Because the intervention was implemented at hospital level, consent was not sought from individual patients. This study design also avoided selection bias and ensured that, unlike in most cardiovascular trials, our study population was representative, comprising low-risk and high-risk individuals, an equal proportion of men and women, patients who presented out-of-hours, and those who were unwell and unlikely to survive. Second, throughout the trial, contemporary and high-sensitivity troponin assays were run simultaneously in plasma that was surplus to requirement, to accurately identify all patients reclassified by high-sensitivity testing during both phases of the trial. Third, we used established regional and national registries^12^, ^15^ to track investigations, treatments, and outcomes in all patients through linkage of electronic health-care records ensuring 100% follow-up in those patients who remained resident in Scotland. Finally, these linked datasets were used to assess all index and primary outcome events in accordance with the Universal Definition of Myocardial Infarction.

We previously showed that lowering the diagnostic threshold with a contemporary troponin assay was associated with a reduction in myocardial infarction or death in those reclassified as having myocardial infarction.^11^ Despite these improvements, several observational studies^17^, ^18^, ^19^ that evaluated high-sensitivity assays have suggested that myocardial infarction is underdiagnosed with contemporary assays and that misdiagnosis is associated with excess mortality.^20^ In this context, we expected that the introduction of a high-sensitivity assay would improve outcomes. However, we observed no difference in the primary or secondary efficacy outcomes within 1 year in patients reclassified with the high-sensitivity assay. This finding was despite the assay identifying a group with similar cardiovascular risk factors as those with more extensive myocardial injury, and despite 236 (13%) of 1771 patients having a subsequent myocardial infarction or cardiovascular death within 1 year.

Several observations could explain our findings. First, only a third of patients who were reclassified by the high-sensitivity assay actually had a diagnosis of type 1 myocardial infarction and would therefore benefit from evidence-based therapies. Second, although patients with myocardial injury or type 2 myocardial infarction are known to have poor outcomes,^21^, ^22^, ^23^ this population is very heterogeneous, and no evidence from randomised trials yet exists to guide treatment in these patients. Third, although new prescriptions for anti-platelet, statin, and beta-blocker therapies doubled and the frequency of coronary angiography tripled in the implementation phase, overall only 1 in 10 patients received an additional secondary preventive drug or underwent angiography. However, many patients reclassified by the high-sensitivity assay were already known to have ischaemic heart disease and were receiving secondary prevention at the time of presentation, which might have attenuated the potential to improve outcomes. Finally, most patients reclassified by the high-sensitivity assay were women, because the sex-specific 99th centile is lower in women than men. Many studies^3^, ^24^ have reported that women are less likely to receive investigations and treatments for coronary heart disease than men, and this could have further attenuated any benefit of implementing the high-sensitivity assay.

Although the duration of stay doubled in those reclassified by the high-sensitivity assay, it was reduced by a third across the trial population. This reduction was because most patients did not have myocardial injury or infarction, and their duration of hospital stay halved. Importantly, implementation might have improved the treating clinician's confidence that myocardial infarction had been ruled out, resulting in an earlier discharge; our findings of reduced hospital stay duration are consistent with a previous study.^25^ Emerging evidence suggests that very low hs-cTnI concentrations at presentation can identify half of all patients as low risk.^12^, ^26^, ^27^ Similar observations have been reported for cardiac troponin T,^28^, ^29^ and risk stratification thresholds below the 99th centile have been incorporated into early rule-out pathways.^30^, ^31^, ^32^, ^33^, ^34^ The 2016 European Society of Cardiology guidelines^35^ recommend the use of pathways that incorporate thresholds below the 99th centile and small changes in cardiac troponin to improve both the rule-in and rule-out of myocardial infarction. Together, these approaches have the potential to improve the efficiency of health-care systems, but prospective randomised controlled trials are ongoing to determine the effectiveness and safety of these pathways and their impact on patient care (such as ClinicalTrials.gov NCT03005158 and Australian New Zealand Clinical Trials Registry ACTRN12615001379505).

To our knowledge, our findings represent the first evidence from a randomised controlled trial that evaluates the recommendations of the Universal Definition of Myocardial Infarction. Despite consistently implementing these guidelines across all sites, use of the hs-cTn I assay did not improve outcomes for patients. In contrast to earlier studies,^11^ in which improvements in assay performance were associated with benefits in reducing the diagnostic threshold from 200 ng/L to 50 ng/L, further reductions identified a heterogeneous group of patients. The recommendation that the 99th centile from a healthy reference population be used to diagnose myocardial infarction is based on expert consensus and observational studies rather than evidence from randomised controlled trials. Registry studies^36^, ^37^, ^38^ suggest that the introduction of a high-sensitivity cardiac troponin T assay was associated with better risk stratification of patients in the Emergency Department and more percutaneous coronary intervention with lower rates of recurrent myocardial infarction, but these studies showed no difference in all-cause mortality. By contrast, we showed that implementation of a high-sensitivity assay did not improve clinical outcomes in our randomised controlled trial despite accurately identifying the group of patients most likely to benefit. This finding raises the question of what is the optimal approach to diagnose myocardial infarction. Should clinical decisions be based on a statistical threshold derived from a reference population, or an approach that acknowledges the continuum of disease and optimises diagnostic accuracy?

There are some study limitations. This was a pragmatic trial, and therefore we had to accept some flexibility in the implementation phase to accommodate shared out-of-hours laboratory services, shared electronic patient records, and site closures (appendix). The proportion of patients reclassified with the high-sensitivity assay was smaller than anticipated from our pilot study^3^ but, given the consistency of our findings across a range of endpoints, it is unlikely a larger trial would have yielded a different result. Hospitals that use lower contemporary assay thresholds would reclassify fewer patients when implementing a high-sensitivity assay, but the effect on subsequent myocardial infarction or death from cardiovascular causes would probably be similar. A previous study^39^ has suggested that higher diagnostic thresholds should be applied in patients with renal impairment, and we did not evaluate this approach in our trial. However, renal function was similar in patients with myocardial injury, whether reclassified by the high-sensitivity assay or identified by the contemporary assay, suggesting that the prevalence of this comorbidity is not the primary explanation for our findings. Finally, further research is required to understand how the changing criteria for the classification of patients with myocardial injury and infarction will affect patient management and clinical outcomes.

In conclusion, we have shown that implementation of a high-sensitivity cardiac troponin I assay prompted reclassification of 1771 (17%) of 10 360 patients with myocardial injury; however, only a third of these patients had a diagnosis of type 1 myocardial infarction, and the incidence of subsequent myocardial infarction or death from cardiovascular causes within 1 year was not affected by use of this assay.

## Data sharing
