# Supplementary material for: High-sensitivity troponin in the evaluation of patients with suspected acute coronary syndrome: a stepped-wedge, cluster-randomised controlled trial
Source: Lancet. 2018 Sep 15;392(10151):919–28. doi: 10.1016/S0140-6736(18)31923-8 (PMC6137538; doi:10.1016/S0140-6736(18)31923-8)
Supplement: Supplementary appendix [file mmc1.pdf]

THE LANCET

Supplementary appendix

This appendix formed part of the original submission and has been peer reviewed. We post it as supplied by the authors.

Supplement to: Shah ASV, Anand A, Strachan FE, et al. High-sensitivity troponin in the evaluation of patients with suspected acute coronary syndrome: a stepped-wedge, cluster-randomised controlled trial. *Lancet* 2018; published online Aug 28. http://dx.doi.org/10.1016/S0140-6736(18)31923-8.

High-sensitivity troponin in the evaluation of patients with suspected acute coronary syndrome: a cluster-randomised controlled trial

Anoop S.V. Shah, P.hD.,[1]* Atul Anand, M.B.Ch.B.,[1]* Fiona E. Strachan, Ph.D.,[1]* Amy V. Ferry, B.Sc.,[1] Kuan Ken Lee, M.D.,[1] Andrew R. Chapman, M.D.,[1] Dennis Sandeman, M.Sc.,[2] Catherine L. Stables, Ph.D.,[1] Philip D. Adamson, Ph.D.,[1] Jack P.M. Andrews,[1] M.D., Mohamed S. Anwar, M.D.,[1] John Hung, M.D.,[1] Alistair J. Moss, M.D.,[1] Rachel O'Brien, B.N., Colin Berry, M.D.,[4] Iain Findlay, M.D.,[5] Simon Walker, D.M.,[6] Anne Cruickshank, M.D.,[7] Alan Reid, M.Sc.,[7] Alasdair Gray, M.D.,[3] Paul O. Collinson, M.D.,[8] Fred S. Apple, Ph.D.,[9] David A. McAllister, M.D.,[10] Donogh Maguire, M.B.Ch.B.,[11] Keith A.A. Fox, M.B.Ch.B.,[1] David E. Newby, D.Sc.,[1] Christopher Tuck, B.Sc.,[12] Ronald Harkess, B.Sc.,[12] Richard A. Parker, M.Sc.,[12] Catriona Keerie, M.Sc.,[12] Christopher J. Weir, Ph.D.,[12] Nicholas L. Mills, M.D.[1]; *on behalf of the High-STEACS Investigators*[†]

[1] BHF Centre for Cardiovascular Science, University of Edinburgh, Edinburgh, UK.
[2] Department of Cardiology, Victoria Hospital Kirkcaldy, Kirkcaldy, UK.
[3] Emergency Medicine Research Group Edinburgh, Royal Infirmary of Edinburgh, Edinburgh, UK.
[4] Institute of Cardiovascular and Medical Sciences, University of Glasgow, Glasgow, UK.
[5] Department of Cardiology, Royal Alexandra Hospital, Paisley, UK.
[6] Department of Clinical Biochemistry, Royal Infirmary of Edinburgh, Edinburgh, UK.
[7] Department of Biochemistry, Queen Elizabeth University Hospital, Glasgow, UK.
[8] Departments of Clinical Blood Sciences and Cardiology, St George's, University Hospitals NHS Trust and St George's University of London, London, UK.
[9] Department of Laboratory Medicine and Pathology, Hennepin County Medical Center & University of Minnesota, Minneapolis, MN, USA
[10] Institute of Health and Wellbeing, University of Glasgow, Glasgow, UK.
[11] Emergency Medicine Department, Glasgow Royal Infirmary, Glasgow, UK.
[12] Edinburgh Clinical Trials Unit, University of Edinburgh, Edinburgh, UK.

*Contributed equally

Corresponding Author:
Professor Nicholas L Mills
BHF/University Centre for Cardiovascular Science
The University of Edinburgh
Edinburgh EH16 4SA
United Kingdom
Telephone: 0044 131 242 6515
E-mail: nick.mills@ed.ac.uk

Table of Contents

Supplementary text ………………………………………………………………. Page 3

Supplementary tables ……………………………………………………………. Page 9

Supplementary figures …………………………………………………………. Page 12

References …………………………………………………………………… Page 13

Randomisation

Block randomisation was used with sites paired based on the expected number of presentations (***Table S1***) and one site randomised to early implementation and the other to late implementation. For pragmatic reasons (shared lab facilities out of hours), the Vale of Leven and Royal Alexandra Hospital, Paisley were grouped and randomised together. This enabled implementation of the high-sensitivity assay to occur on the same date at both sites and allowed the same lab processes to be followed at both sites. The randomisation sequence was generated by a programmer at the Edinburgh Clinical Trials Unit who was not otherwise involved in the study using computer generated pseudo-random numbers.

Implementation support

To support implementation, we provided written educational material and presentations at each site, training for clinical and laboratory staff, and we updated the electronic patient record to highlight the change in assay and diagnostic thresholds. Educational material on the new assay and decision thresholds was presented at each Emergency Department handover (twice daily) during the implementation phase to ensure wide coverage of staff on all shift patterns. This was reinforced by specialist chest pain nurses who received detailed training prior to implementation and who support Emergency Department clinicians in the assessment of patients with suspected acute coronary syndrome. Key details from the educational presentation formed a one-page reference guide that was posted within each department and online in the hospital guidelines portal. This information was also presented to the wider hospital teams in medical grand round presentations prior to implementation, and circulated to all general practitioners. Every high-sensitivity cardiac troponin result reported in the electronic health record during the implementation phase was accompanied with guidance

notes outlining the new assay, reporting units and thresholds. Laboratory staff also received training to ensure any queries directed to the laboratory were dealt with consistently. Finally, the research team included senior cardiologists, emergency physicians, and cardiology nurses who are clinically active within each of the hospital clusters; education was therefore reinforced at a local level by these clinical leaders throughout the implementation phase.

Outcomes

All in-hospital and community deaths, and all hospital admissions are recorded on the Register of Deaths in Scotland and the Scottish Morbidity Record (SMR) respectively. It is a statutory requirement that any deaths occurring in Scotland, or outwith Scotland but within the United Kingdom are entered on the Register of Deaths in Scotland within eight days of death. As such, this registry is 100% complete for the study population, which was restricted to those resident in Scotland. This makes an assumption that patients did not emigrate in the year following enrollment. However, the Scottish population is very stable, with low levels of emigration outwith the United Kingdom.

The TrakCare software application (InterSystems Corporation, Cambridge, MA, USA) is an electronic patient record system used at all participating sites, which provided clinical data for all subsequent hospital admissions. All attendances across any participating hospital where cardiac troponin was measured and the hs-cTnI concentration was >99th centile were reviewed and the diagnosis adjudicated. We used the same approach to adjudication as for the index hospital episode with the panel blinded to all cardiac troponin measurements during the index episode and to the study phase.

The primary outcome was myocardial infarction (type 1 or type 4b) or cardiovascular death at 1 year. Secondary efficacy end-points include myocardial infarction, unplanned coronary revascularisation, cardiovascular death, cardiac death, all-cause death, duration of stay, hospitalisation for heart failure, and ischaemic stroke. Secondary safety end-points include major haemorrhage, unplanned hospitalisation excluding acute coronary syndrome, and non-cardiovascular death.

Unplanned coronary revascularisation was defined as any urgent or emergency percutaneous coronary intervention or coronary artery bypass grafting following discharge. International Classification of Disease (ICD)-10 codes from the Scottish Morbidity Record were used to define hospitalisation for heart failure (I50) and ischaemic stroke (I63, I65, or I66). Bleeding was defined according to the Bleeding Academic Research Consortium (BARC) definition using ICD-10 and OPCS codes to classify each bleeding event as previously described (1, 2). Major haemorrhage was defined as BARC type 3 or type 5. Unplanned hospitalisation excluding acute coronary syndrome was defined as any hospital attendance or admission excluding type 1 or type 4b myocardial infarction at 30 days.

The duration of stay was derived from a common electronic patient record system used across all participating sites (TrakCare, InterSystems Corporation, Cambridge, MA, USA) and was calculated from the admission and discharge date and time to the nearest minute.

The decision to perform coronary angiography was made by the attending cardiologist taking into consideration all aspects of the patients presentation including cardiac troponin concentrations.

The Scottish national community drug-prescribing database of ISD in NHS Scotland maintains a detailed record of all prescriptions dispensed in the community, which are linked to individual patient identifiers. Alterations in cardiovascular therapies following the index hospital episode were determined by comparison to baseline.

The Scottish Index of Multiple Deprivation (SIMD) identifies areas where the highest concentration of deprivation exists in Scotland. SIMD 2012 combines 38 different indicators covering seven different dimensions of deprivation including income, employment, health, education, housing, access to services and crime (3). The SIMD is derived for each individual in the trial population from the postcode at their address of residence.

We calculated Global Registry of Acute Coronary Events (GRACE) risk scores and stratified patients as low risk (<1% risk of in-hospital mortality) or intermediate–high risk (≥1% risk of in-hospital mortality (4).

Reporting

We report all primary and secondary efficacy outcomes in the pre-specified statistical analysis plan. However, two of the eleven secondary end-points specified in the original trial protocol are not reported. During the conduct of the trial it became clear that ‘minor haemorrhage’ and ‘cardiovascular death excluding acute coronary syndrome’ would be difficult to define from routine healthcare data. Following discussion with our trial steering committee, these secondary endpoints were not included in the pre-specified statistical analysis plan and are therefore not presented in the manuscript.

Site Closures

In order to accommodate the closure of the Western Infirmary and Victoria Infirmary during the implementation phase of the study, and subsequent redirection of patients in their catchment areas to the new Queen Elizabeth University Hospital on the site of the Southern General Hospital, patients were analysed according to the site at which they were treated. The Queen Elizabeth University Hospital was considered a continuation of the Southern General Hospital site.

Sample Size

Using previous data from the Royal Infirmary of Edinburgh (5), we estimated that patients reclassified by the high-sensitivity assay would experience an event rate of 13% for the primary outcome of subsequent myocardial infarction or cardiovascular death. We originally planned that 10 sites (clusters) would include patients during three 6-month phases: validation (standard care), randomization (early or late introduction of the intervention) and implementation (intervention). For each site the difference in proportions of primary outcome events between standard care and intervention will be approximately normally distributed, each with a standard deviation that depends on the number of patients recruited by that site and the primary outcome event rate under standard care (assumed to be 13%). During a pilot phase (6), detailed power calculations, based on 1,000 simulations per scenario, were performed based on the anticipated proportion of patients who would be reclassified by the high-sensitivity assay. Power for a reclassification rate ranging from 6% to 9%, was 74% to 85% for an absolute risk reduction of 4·4%. Power was virtually unchanged when varying the ICC value from 0·05 to 0·10, as would be expected in a stepped wedge design such as this in which relatively few clusters recruit a large number of patients per site.

Secondary analysis

Survival times were analysed using a Cox-proportional hazards model similar to the generalised logistic regression model for the primary analysis, fitting hospital site as a random effect and age (centred on the analysis population mean), sex and SIMD quintile as patient-level fixed effects. The effect of implementing the high-sensitivity assay was estimated by the hazard ratio and its 95% confidence interval. In patients reclassified by the high-sensitivity assay, the primary outcome occurred in 14·6% (105/720) and 12·5% (131/1,051) during the validation and implementation phases respectively (hazard ratio 1.12, 95% confidence interval 0.79 to 1.57, P=0.53).

Data Sharing

The High-STEACS trial makes use of multiple routine electronic health care data sources that are linked, deidentified and held in our national safe haven, which is accessible by approved individuals who have undertaken the necessary governance training. Summary data can be made available upon request to Nicholas Mills (nick.mills@ed.ac.uk).

Table S1. Number of Hospitalizations with Chest Pain and Myocardial Infarction at Participating Sites in 2010

	Chest pain	Myocardial infarction	Assay platform available	Diagnostic threshold for contemporary cTnI assay
Vale of Leven General Hospital	455	132	Yes	40 ng/L
Inverclyde Royal Hospital	980	135	Yes	40 ng/L
Royal Alexandra Hospital	1,371	178	Yes	40 ng/L
Glasgow Royal Infirmary	1,791	227	Yes	40 ng/L
Victoria Infirmary	1,670	315	Yes	40 ng/L
Southern General Hospital	1,274	163	Yes	40 ng/L
Western Infirmary	1,867	187	Yes	40 ng/L
Western General Hospital	379	103	Yes	50 ng/L
St John's Hospital	1,001	211	Yes	50 ng/L
Royal Infirmary of Edinburgh	2,051	864	Yes	50 ng/L

Source: Scottish Morbidity Record from eDRIS NHS Scotland for 2010 linked on 26 February 2011 using the following ICD-10 codes: chest pain (R7) and myocardial infarction (I21, I22). cTnI = cardiac troponin I

Table S2. Characteristics of Trial Participants Stratified by both Troponin Concentration and Phase

	No myocardial injury		Myocardial injury			
			Reclassified by hs-cTnI assay		Identified by cTnI assay	
	Validation	Implementation	Validation	Implementation	Validation	Implementation
No. of participants	14,862 (39)	23,060 (61)	720 (41)	1,051 (59)	3,396 (40)	5,193 (60)
Age, years	59±17	58±17	76±13	74±14	71±15	70±15
Women	7,042 (47)	10,529 (46)	612 (85)	858 (82)	1,460 (43)	2,061 (40)
Presenting complaint*						
Chest pain	8,677 (83)	19,414 (84)	373 (68)	701 (67)	1,643 (68)	3,732 (72)
Dyspnoea	383 (4)	724 (3)	66 (12)	136 (13)	299 (12)	567 (11)
Palpitation	291 (3)	700 (3)	19 (3)	53 (5)	76 (3)	130 (3)
Syncope	567 (5)	1242 (5)	42 (8)	83 (8)	220 (9)	341 (7)
Other	490 (5)	968 (4)	50 (9)	78 (7)	180 (7)	422 (8)
Past medical history						
Myocardial infarction	1,353 (9)	1,482 (6)	97 (13)	122 (12)	519 (15)	641 (12)
Ischemic heart disease	3,861 (26)	4,594 (20)	300 (42)	345 (33)	1,231 (36)	1,581 (30)
Cerebrovascular disease	877 (6)	1,038 (5)	88 (12)	122 (12)	367 (11)	457 (9)
Diabetes mellitus	961 (6)	1,079 (5)	95 (13)	123 (12)	516 (15)	744 (14)
Previous revascularisation						
PCI	1,204 (8)	1,540 (7)	66 (9)	89 (8)	311 (9)	472 (9)
CABG	238 (2)	296 (1)	18 (3)	22 (2)	93 (3)	115 (2)
Medications at presentation						
Aspirin	4,231 (28)	5,231 (23)	300 (42)	368 (35)	1,309 (39)	1,724 (33)
Dual anti-platelet therapy†	583 (4)	520 (2)	41 (6)	47 (4)	219 (6)	195 (4)
Statin	5,994 (40)	8,112 (35)	403 (56)	557 (53)	1,794 (53)	2,506 (48)
ACE inhibitor or ARB	4,681 (31)	6,604 (29)	317 (44)	445 (42)	1,451 (43)	2,120 (41)
Beta-blocker	4,183 (28)	5,383 (23)	285 (40)	373 (35)	1,271 (37)	1,678 (32)

Oral anti-coagulant‡	938 (6)	1,220 (5)	94 (13)	144 (14)	379 (11)	478 (9)
Electrocardiogram§						
Normal	-	-	212 (43)	380 (43)	780 (34)	1,300 (31)
Myocardial ischemia	-	-	67 (14)	127 (14)	720 (31)	1,596 (38)
ST-segment elevation	-	-	9 (2)	23 (3)	255 (11)	711 (17)
ST-segment depression	-	-	51 (10)	74 (8)	409 (18)	794 (19)
Left bundle branch block	-	-	10 (2)	20 (2)	53 (2)	104 (2)
T-wave inversion	-	-	85 (17)	107 (12)	399 (17)	686 (16)
Physiological parameters						
Heart rate, beats per minute	-	-	84±26	87±27	87±26	85±26
Systolic blood pressure, mmHg	-	-	143±27	143±28	137±29	138±29
GRACE risk score	-	-	142±30	141±33	144±37	144±40
Hematology and clinical chemistry						
Hemoglobin, g/L	137±20	138±20	~~126±22~~	~~124±22~~	132±26	133±25
eGFR, mL/min	56±10	55±11	~~47±15~~	~~48±15~~	48±16	47±16
Peak hs-cTnI, ng/L	3 [1-6]	3 [1-6]	25 [20-35]	27 [21-38]	215 [62-1,570]	398 (87-3,584)
Serial hs-cTnI testing‖	7,312 (49)	8,716 (38)	357 (50)	667 (63)	2,352 (69)	3,607 (69)

Presented as No. (%), mean±SD or median [inter-quartile range]. Abbreviations: ACE = angiotensin converting enzyme; ARB = angiotensin receptor blockers; eGFR = estimated glomerular filtration rate; CABG = coronary artery bypass grafting; GRACE = Global Registry of Acute Coronary Events; PCI = percutaneous coronary intervention. *Presenting symptom was missing in 4,466 (12%) patients. [†]Two medications from aspirin, clopidogrel, prasugrel or ticagrelor. ‡Includes warfarin or novel oral anti-coagulants. § Electrocardiographic data was available in 78% and 75% of reclassified and identified patients respectively. ‖ Serial testing is defined as two or more tests within 24 hours from presentation.

Figure S1. CONSORT Diagram of the Trial and Populations

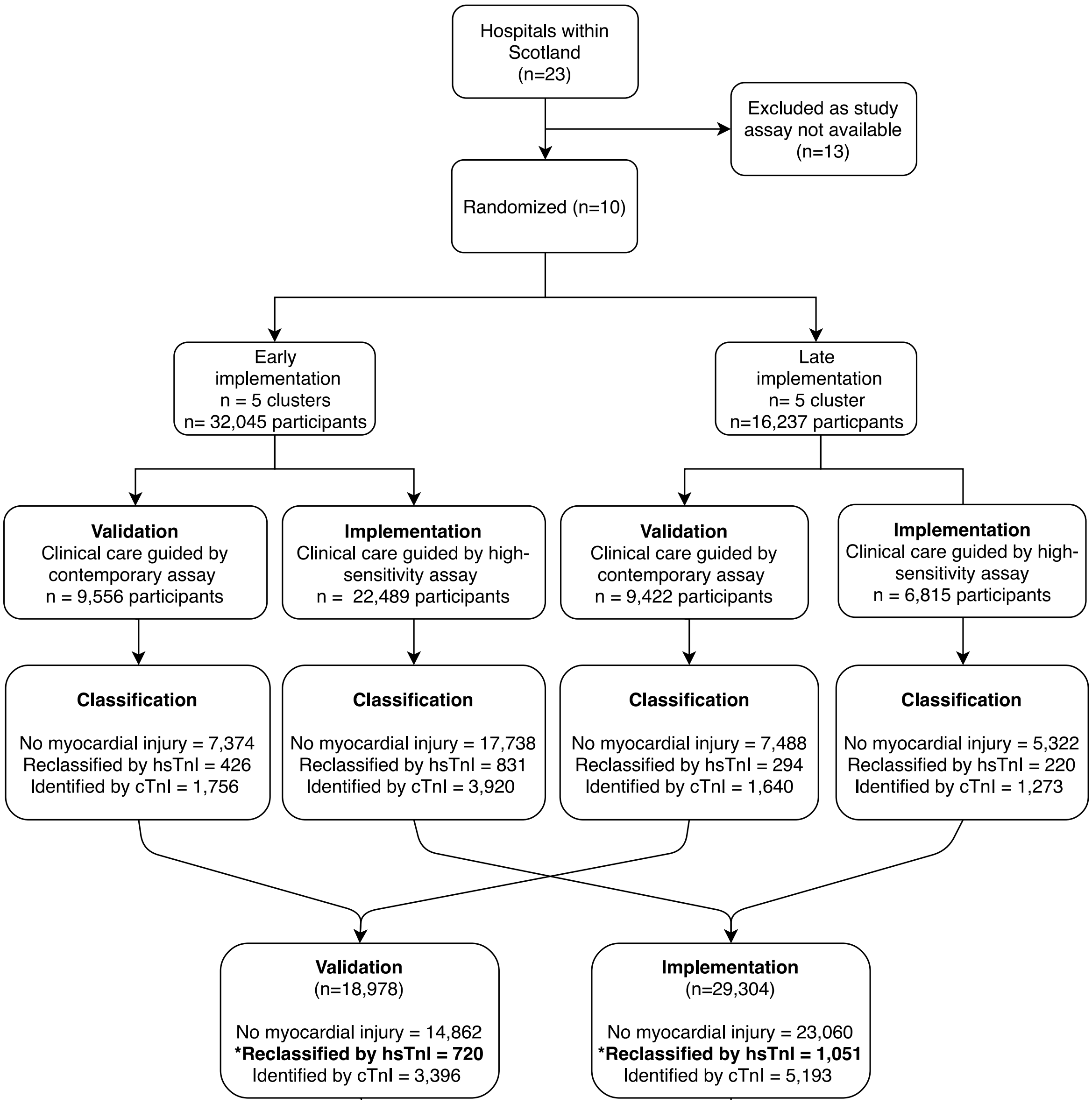

References

1. Mehran R, Rao SV, Bhatt DL, *et al.* Standardized bleeding definitions for cardiovascular clinical trials: a consensus report from the Bleeding Academic Research Consortium. *Circulation*. 2011; 123(23): 2,736–47.

2. Denaxas SC, George J, Herrett E, *et al*. Data resource profile: cardiovascular disease research using linked bespoke studies and electronic health records (CALIBER). *Int J Epidemiol* 2012; 41(6): 1,625–38.

3. The Scottish Index of Multiple Deprivation. Scottish Government. Accessed August 1st 2018, www.gov.scot/Topics/Statistics/SIMD.

4. CB Granger, RJ Goldberg, O Dabbous, *et al.* Predictors of hospital mortality in the global registry of acute coronary events. *Arch Intern Med* 2003; 163: 2,345–53.

5. Mills NL, Churchhouse AM, Lee KK, *et al.* Implementation of a sensitive troponin I assay and risk of recurrent myocardial infarction and death in patients with suspected acute coronary syndrome. *JAMA* 2011; 305(12): 1210–6.

6. Shah ASV, Griffiths M, Lee KK, *et al*. High sensitivity cardiac troponin and the under-diagnosis of myocardial infarction in women: prospective cohort study. *BMJ* 2015; 350: g7873.
